# Epidemiology of *Rhodotorula*: An Emerging Pathogen

**DOI:** 10.1155/2012/465717

**Published:** 2012-10-02

**Authors:** Fernanda Wirth, Luciano Z. Goldani

**Affiliations:** Section of Infectious Diseases, Hospital de Clínicas de Porto Alegre, Universidade Federal do Rio Grande do Sul Ramiro Barcelos 2350, 90640-002 Porto Alegre, RS, Brazil

## Abstract

This is an updated paper focusing on the general epidemiological aspects of *Rhodotorula* in humans, animals, and the environment. Previously considered nonpathogenic, *Rhodotorula* species have emerged as opportunistic pathogens that have the ability to colonise and infect susceptible patients. *Rhodotorula* species are ubiquitous saprophytic yeasts that can be recovered from many environmental sources. Several authors describe the isolation of this fungus from different ecosystems, including sites with unfavourable conditions. Compared to *R. mucilaginosa*, *R. glutinis* and *R. minuta* are less frequently isolated from natural environments. Among the few references to the pathogenicity of *Rhodotorula* spp. in animals, there are several reports of an outbreak of skin infections in chickens and sea animals and lung infections and otitis in sheep and cattle. Most of the cases of infection due to *Rhodotorula* in humans were fungemia associated with central venous catheter (CVC) use. The most common underlying diseases included solid and haematologic malignancies in patients who were receiving corticosteroids and cytotoxic drugs, the presence of CVC, and the use of broad-spectrum antibiotics. Unlike fungemia, some of the other localised infections caused by *Rhodotorula*, including meningeal, skin, ocular, peritoneal, and prosthetic joint infections, are not necessarily linked to the use of CVCs or immunosuppression.

## 1. Introduction

*Rhodotorula* is a common environmental yeast that is found in air, soil, lakes, ocean water, milk, and fruit juice. *Rhodotorula* species, part of the Basidiomycota phylum, colonise plants, humans, and other mammals. The genus *Rhodotorula* includes eight species, of which *R. mucilaginosa, R. glutinis, and R. minuta* are known to cause disease in humans [1]. *Rhodotorula* produces pink to red colonies and blastoconidia that are unicellular lacking pseudohyphae and hyphae. Several authors have isolated *Rhodotorula* in different ecosystems and environments as well as described infections in animals. *Rhodotorula* spp. have been recognised as emerging yeast pathogens in humans in the last two decades. While no cases of *Rhodotorula* infection were reported in the medical literature before 1985, the number of infections increased after that time, most likely because of the wider use of intensive treatments and central venous catheters (CVCs) [2].

This is an updated concise paper focusing on the general epidemiological aspects of *Rhodotorula* in humans, animals, and the environment. 

## 2. *Rhodotorula* in the Environment and Nonhumans

*Rhodotorula* species are ubiquitous saprophytic yeasts that can be recovered from many environmental sources. This yeast has a strong affinity for plastic, having been isolated from various medical equipments, such as dialysis equipment, fibre-optic bronchoscopes, and other environmental sources, including shower curtains, bathtubs, and toothbrushes [3–5].

Several authors describe the isolation of this fungus from different ecosystems, including sites with unfavourable conditions, such as the depths of the Baltic Sea, the high-altitude Lake Patagonia, the soil and vegetation of Antarctica and aquatic, hypersaline, and high-temperature environments such as the Dead Sea (Israel), Lake Enriquillo (Dominican Republic), the Great Salt Lake (USA), and beaches located in northern Brazil. In this study, *R. mucilaginosa* was the third most isolated yeast in seawater. Two other studies have reported the occurrence of *Rhodotorula* species in marine waters polluted by household waste [6–10].

*R. mucilaginosa* is commonly isolated from foods and beverages. Several studies have reported the presence of *R. mucilaginosa* in peanuts, apple cider, cherries, fresh fruits, fruit juice, cheese, sausages, edible molluscs, and crustaceans [11–17]. Although the consumption of food contaminated with yeast may not have a direct role in causing opportunistic infections, there is growing concern that food may be an underestimated source of environmental pathogens [18]. Compared to *R. mucilaginosa, R. glutinis *and* R. minuta* are less frequently isolated in natural environments. These species have been detected in air, seawater (including deep environments), freshwater, and goat's milk [19–21]. Environmental studies have documented the presence of *Rhodotorula* sp. in tropical fruits, sugar cane, and shrimp in the waters of Sepetiba Bay in Brazil [22–24]. Tomsíková reported, *Rhodotorula* sp. contamination of food that is provided to immunocompromised patients in hospitals. [25]. In addition, environmental monitoring of yeasts in specific areas of two tertiary local hospitals revealed the presence of *Rhodotorula* species in a substantial amount of air samples [26]. As a direct consequence of the wide exposure to *Rhodotorula* in the hospital environment, patients who have a depressed immune system can develop Rhodotorulosis, causing a variety of systemic infections. In fact, *Rhodotorula* spp. is the most common microorganism isolated from the hands of hospital employees and patients [27]. Further studies are needed to clarify the role of food contamination by *Rhodotorula* and the development of opportunistic fungal infections. These future studies should focus on the survival and growth of *Rhodotorula* in the gastrointestinal system and its potential ability to transfer from the gastrointestinal tract to bloodstream and should seek to better understand the ecology of *Rhodotorula* in hospitals and healthcare environments [28]. In this aspect, *Rhodotorula* spp. have been isolated from stool samples, indicating that these yeasts can survive in the extreme conditions of the gastrointestinal tract, and it is still uncertain whether *Rhodotorula* is capable of passing from the gastrointestinal tract into the bloodstream [29, 30]. 

 Among the few references about the pathogenicity of *Rhodotorula* spp. in animals, there are several reports of an outbreak of skin infections in chickens and a report of a lung infection in sheep, both caused by *R. mucilaginosa* [31, 32]. *Rhodotorula* was reported as the causative agent of epididymitis, skin lesions in a sea lion, and dermatitis in a cat that had crusted lesions and mastitis [33–36]. Interestingly, this fungus can also be found in pools where sea animals are kept in captivity [37]. Duarte et al. have shown the presence of fungi in the ear canal of 45 cattle with external parasitic otitis. The 45 cultures in Sabouraud dextrose medium revealed the growth of the genus *Malassezia* in 31 (68.9%) of the 45 cultures, seven (15.5%) yeasts of the genus Candida, five (11.1%) *R. mucilaginosa*, and two (4.4%) fungi of the genus *Aspergillus* [38]. Some authors have reported the *Rhodotorula* genus as a colonising agent in the oropharynx and cloaca of ostriches, in faecal samples and the cloaca of wild birds and pigeons in urban and suburban areas, in the ear canals of adult cattle with parasitic otitis, in healthy rhesus monkeys, genital tract of healthy female camels, and in healthy cats [39–44].

 Animal models have been used to study the mechanisms of pathogenesis of different human fungal diseases. Recently, Wirth and Goldani conducted the first experimental study in an animal model of disseminated *Rhodotorula* infection described in the literature [45]. Organs such as lungs, spleen, and especially the liver were the most affected organs presented severe degree of infection. Considering that the animals were highly immunocompromised, histopathology of the involved organs revealed few epitelioidcellsand multinuclear giant cells in association with abundant yeast forms with occasional granuloma formation.

## 3. *Rhodotorula* in Humans

Previously considered nonpathogenic, *Rhodotorula* species have emerged as opportunistic pathogens with the ability to colonise and infect susceptible patients. Recent studies have demonstrated that the incidence of fungemia caused by *Rhodotorula* was between 0.5% and 2.3% in the USA [46, 47] and Europe [48]. Most cases of infection with *Rhodotorula* fungemia are associated with central catheters in patients with haematologic malignancies. [46, 47, 49–52]. Considering that *Rhodotorula* is an ubiquitous and saprophytic fungus, the isolation of *Rhodotorula* from nonsterile human sites, especially from the mucous membranes, has often been of questionable clinical significance. Localised infections without fungemia including endophthalmitis, onychomycosis, meningitis, prosthetic joint infections, and peritonitis (usually associated with continuous peritoneal dialysis) have been reported in immunocompromised and immunocompetent patients.

 The first report of fungemia caused by *Rhodotorula* was made by Louria et al. in 1960 [47]. Subsequently, an increasing number of cases have been published, especially in the last two decades. However, this increase may be a publication bias after recognition of *Rhodotorula* as a pathogen [52]. Another possible explanation is the dramatic expansion of new treatment modalities related to critical care medicine and transplantation. 

 From 1970 until 1985, no cases of *Rhodotorula* infection were reported in haematological patients, but the number of cases of *Rhodotorula* infection in these patients increased after 1985. The increase of *Rhodotorula* fungemia related to catheters was associated with an increase of more aggressive treatment modalities, which include intensive care units admissions, use of central venous catheters, short- and long-term parenteral nutrition, broad-spectrum antibiotics, organ transplants, and chemotherapy [51]. Most of cases reported in the literature date back to after 1994, when CVCs and intensive therapies were widely available.  Table 1 shows a summary of reported cases of fungemia related to CVC use between 2000 and 2011 [46–68]. In all cases listed, the patients were using CVC, short- or long-term CAPD, and umbilical catheter. Zaas et al. [52] published a large number of cases of CVC-related fungemia by *Rhodotorula* spp. that occurred in a USA hospital [52]. The most prevalent species was *R. mucilaginosa*, followed by *R. glutinis*. Most of the patients had an underlying disease, such as congenital heart disease, AIDS, cancer, or chronic intestinal disease, and two patients were transplant recipients (one lung and one bone marrow). Two patients were neutropenic at the time of the development of fungemia, and five patients were receiving parenteral nutrition. All the patients received antifungal treatment. Only in three patients the CVC was not removed. There were no reports of death or relapse of infection.

Perniola et al. [60] reported four cases of CVC-related fungemia by *R. mucilaginosa* in a neonatal intensive care unit (NICU) in an Italian hospital [60]. All the newborns infected with fungemia by *R. mucilaginosa* were premature; three had bacteremia prior to fungemia, and 3 received prophylactic fluconazole. All 4 neonates had venous access (a CVC, an umbilical venous catheter, or both) since birth, but early removal or replacement of the catheter followed by confirmation of sepsis by *R. mucilaginosa* was possible in only two newborns. Blood cultures performed at the end of antifungal therapy were negative. Another retrospective study reviewed the demographics, risk factors, treatment, and outcome of seven patients with *Rhodotorula* fungemia over the years from 2002 to 2005 in a Brazilian hospital [46]. Risk factors included solid and haematologic malignancies in patients who were receiving corticosteroids and cytotoxic drugs, the presence of CVCs, and the use of broad-spectrum antibiotics. Three of the seven patients died, with an overall mortality rate of 42%. The result was favourable for patients who had just had the CVC removed. Duboc de Almeida et al. described 25 cases of fungemia by *R. Mucilaginosa* [48]. The majority of patients had a CVC, and 10 patients (40%) had undergone bone marrow transplantation. Amphotericin B deoxycholate was the most commonly antifungal used, and the CVC was removed in 89.5% of patients. Four (17%) patients died [55].

 In a recent paper covering the cases of fungemia by *Rhodotorula* spp. associated with catheters between the years 1966 and 2006, Tuon et al. analysed 66 patients with *Rhodotorula* fungemia. *R. mucilaginosa* was responsible for most of the cases, followed by *R. glutinis* [50]. The most prevalent underlying diseases were haematologic malignancies and solid tumours. AIDS, chronic renal failure, cirrhosis, and gastrointestinal disorders as well as the use of CVCs for parenteral nutrition were also considered predisposing factors.

 A recent literature review published in 2010 by García-Suárez et al. analysed 29 cases of *Rhodotorula* fungemia in patients with haematological disorders [51]. This study showed that 100% of patients who developed fungemia by *Rhodotorula* had some form of central venous access, such as a Hickman catheter. In 2008, Tuon and Costa performed the first systematic review of infections caused by *Rhodotorula* in 128 patients [49]. The authors analysed all papers about *Rhodotorula* infections published until January 2006 [46]. The most common *Rhodotorula* species found by the authors was *R. mucilaginosa*, followed by *R. glutinis* and *R. minuta*. Immunosuppression was found in 40% of patients, and the most common underlying condition associated with *Rhodotorula* infection was the use of CVCs. In a recent paper, Spiliopoulou et al. described a patient who developed *Rhodotorula* fungemia in an intensive care unit. The authors reviewed the risk factors associated with the development of disseminated *Rhodotorula* infection published in several reports including presence of central venous catheters, solid organ neoplasm, abdominal surgery, and administration of antibiotics. In addition, the authors pointed out that *Rhodotorula* is reliably resistant to fluconazole and echinocandins [69]. On the other hand, *in vitro* susceptibility studies revealed that *Rhodotorula* is generally susceptible to amphotericin B and flucytosine.

Unlike fungemia, some of the other infections caused by *Rhodotorula* were not necessarily linked to the use of CVCs or an underlying disease.  Table 2 lists a summary of cases of localized *Rhodotorula* infection that did not cause fungemia occurring between the years 2000 and 2011 [70–90]. Meningitis and endophthalmitis by *Rhodotorula* species have been reported as nosocomial infections especially in human immunodeficiency virus- (HIV-) infected persons [70–78]. Prosthetic joint infections caused by *Rhodotorula* sp. have been reported in an HIV-infected patient and patients without any known immunosuppression [79–81]. Goyal et al. described a case of infection caused by *R. mucilaginosa* that had been unionised as a fracture of the femur (the femoral nonunion). The patient was treated with amphotericin B and required a bone graft [79]. Savini et al. reported a similar case, but the patient was seropositive for HIV and had fractured their left femur [80]. The infection manifested as a chronic coxitis after the patient had undergone surgery for internal fixation. Antifungal therapy was performed using liposomal amphotericin B, which eradicated the infection, and a surgical replacement of the femoral prosthesis was indicated. Peritonitis caused *Rhodotorula* species which have been reported in patients undergoing continuous ambulatory peritoneal dialysis [82–86]. Most of the patients were successfully treated with amphotericin B, and fluconazole was continued after catheter removal. 

The first case of onychomycosis caused by *R. mucilaginosa* was described by Cunha et al., which shows that these yeasts should also be considered as primary agents that can cause opportunistic onychomycosis [87]. The patient was immunocompetent, and the onychomycosis affected the nail of the hallux. In addition to aortic homograft endocarditis, dermatitis, oral ulcers, and lymphadenitis caused by *Rhodotorula* sp. have been reported in the literature [88–91]. 

## 4. Conclusions

*Rhodotorula* species are ubiquitous saprophytic yeasts that can be recovered from many environmental sources. *R. mucilaginosa* is commonly isolated in foods and beverages. Several studies have reported the presence of *R. mucilaginosa* in peanuts, apple cider, cherries, fresh fruits, fruit juice, cheese, sausages, edible molluscs, and crustaceans. *Rhodotorula* was reported as the causative agent in some papers, including dermatitis in sea lions, chickens, and cats, and lung infections and otitis in sheep and cattle. This fungus can also be found in pools where sea animals are kept in captivity. Previously considered nonpathogenic, *Rhodotorula* species have emerged as opportunistic pathogens with the ability to colonise and infect susceptible patients. *Rhodotorula* in humans primarily cause bloodstream infections that are associated with central venous catheter (CVC) use. Risk factors include solid and haematologic malignancies in patients who receive corticosteroids and cytotoxic drugs, the presence of CVCs, and the use of broad-spectrum antibiotics. Unlike fungemia, localised infections caused by *Rhodotorula*, including skin, ocular, meningeal, prosthetic joint, and peritoneal infections, are not necessarily linked to the use of CVCs or an immunosuppression.

## Figures and Tables

**Table 1:** Summary of reports of *Rhodotorula* fungemia (2000–2011).

Reference	Number of described cases	Species	Underlying disease
[46]	7	*R. mucilaginosa *	Hematological-solid malignancies
[52]	10	*R.mucilaginosa *	SBD; LT; AIDS
		*R.glutinis *	
[48]	25	*R. mucilaginosa *	Hematological-solid malignancies; LC; SCA; SBD; CLD
[53]	2	*R. glutinis/R. mucilaginosa *	Lymphoma; solid tumor
[54]	1	*R. mucilaginosa *	Lymphoma
[55]	1	*R. mucilaginosa *	Lymphoma
[56]	2	*R. mucilaginosa *	Acute myeloid leukemia
[57]	1	*R. glutinis *	Nasopharyngeal carcinoma
[58]	1	*R. mucilaginosa *	SBD
[59]	3	*Rhodotorula *spp.	Hematological-solid malignancies
[60]	4	*R. mucilaginosa *	Prematurity
[61]	1	*R. mucilaginosa *	Acute myeloid leukemia
[62]	1	*R. mucilaginosa *	SCA
[63]	2	*R. glutinis *	Broad spectrum antibiotics
[64]	1	*R. glutinis *	Systemic lupus erythematous
[65]	1	*R. glutinis *	Solid organ transplant
[66]	1	*R. glutinis *	Liver cirrhosis
[67]	1	*R. glutinis *	Acute lymphoid leukemia
[68]	1	*R. mucilaginosa *	MS/BMT
[69]	1	*R. mucilaginosa *	Multiple abdominal surgeries; ovarian cancer; bowel necrosis

BMT: bone marrow transplant; CLD: congenital liver disease; LC: liver cirrhosis; LT: lung transplant; MS: myelodysplastic syndrome; SBD: short bowel disease; SCA: sickle cell anemia; SOT: solid organ transplant.

**Table 2:** Summary of reports of *Rhodotorula* infections other than fungemia from 2000 to 2011.

Reference	Species	Underlying disease	Infection site	Outcome
[70–73]	*R. rubra; R. minuta *	HIV; HCV; NU	Endophthalmitis; keratitis	Alive
[74–78]	*R. rubra; R. glutinis; R. mucilaginosa *	HIV; NU	Meningitis	Alive/Death
[79–81]	*R. mucilaginosa; R. minuta *	HIV; NU	Prosthetic joint	Alive
[82–86]	*R. mucilaginosa *	HIV, SOT; chronic renal failure	Peritonitis	Alive/Death
[87]	*R. mucilaginosa *	NU	Onychomycosis	Alive
[88, 89]	*R. mucilaginosa *	HIV,	Oral ulcers, dermatitis	Alive
[90]	*R. mucilaginosa *	NU	Arotic homograft endocarditis	Alive
[91]	*R. mucilaginosa *	HIV	Lymphadenitis	Alive

HCV: chronic hepatitis C; NU: no underlying disease described; SOT: solid organ transplant.
